# Clinical Trial With a Depigmented, Polymerized Mite Mixture Extract at Maximum Concentrations

**DOI:** 10.1002/iid3.70090

**Published:** 2024-12-19

**Authors:** Carmen Vidal, Laura Romero, Sara Lopez‐Freire, Francisco Carballada‐Gonzalez, José Carlos Garcia‐Robaina, Teresa Gonzalez‐Fernandez, Paula Mendez‐Brea, Eva Nieto, Mónica Ruiz‐Garcia

**Affiliations:** ^1^ Complejo Hospitalario de Santiago Santiago de Compostela Spain; ^2^ Hospital Lucus Augusti Lugo Spain; ^3^ Hospital Universitario de La Candelaria Santa Cruz de Tenerife Spain; ^4^ Medical Affairs and Clinical Department LETI Pharma S.L.U. Madrid Spain

**Keywords:** allergic asthma, allergic rhinitis, blomia tropicalis, dermatophagoides pteronyssinus, house dust mites, lepidoglyphus destructor, maximum extract concentrations safety

## Abstract

**Background:**

Efficacy of allergen immunotherapy is dose‐dependent; however, high doses of allergen may imply a greater risk of adverse reactions.

**Objective:**

To assess the safety and tolerability of subcutaneous immunotherapy (SCIT) with mixtures of mite allergen extracts, *Dermatophagoides pteronyssinus*/*Blomia tropicalis* (Dpt/Bt) and *Dermatophagoides pteronyssinus*/*Lepidoglyphus destructor* (Dpt/Ld) at maximum concentrations, in adult patients with allergic rhinitis or rhinoconjunctivitis, and controlled allergic asthma due to a clinically relevant sensitisation to these mites.

**Methods:**

An open‐label, noncontrolled, nonrandomised, phase IIb clinical trial was carried out in three hospitals in Spain between September 2014 and May 2018. Patients received SCIT of either Dpt/Bt (100/1000 DPP/mL) or Dpt/Ld (100/100 DPP/mL) in two phases: a rush build‐up phase on the first day (0.2 mL and 0.3 mL with a 30‐min interval) and a monthly maintenance phase administration (0.5 mL) up to 48 months.

**Results:**

Forty patients were recruited for the study, seven allocated to the Dpt/Bt group and 33 to the Dpt/Ld. None experienced immediate or delayed systemic Grade ≥ 2 reactions (EAACI classification) (systemic reactions were mostly Grade 1) nor died during the study. Local reactions were mostly mild (0‒10 cm). Thirty‐nine patients (97.5%) experienced at least one adverse event (AE). Of the 283 reported AEs, eight (2.8%) were systemic reactions experienced by six (15%) subjects and 14 (4.9%) were local reactions sustained by ten (25%) subjects.

**Conclusions:**

SCIT treatment of patients with allergic rhinitis or rhinoconjunctivitis and controlled asthma with mixtures of Dpt/Bt and Dpt/Ld allergen extracts at maximum concentrations showed a favourable safety profile.

## Graphical abstract

It is an open‐label, noncontrolled, nonrandomised, phase IIb clinical trial. Patients received SCIT of either Dpt/Bt (100/1000 DPP/mL) or Dpt/Ld (100/100 DPP/mL). The treatment of patients with allergic rhinitis or rhinoconjunctivitis and controlled asthma with mixtures of Dpt/Bt and Dpt/Ld at maximum concentrations showed a favourable safety profile.

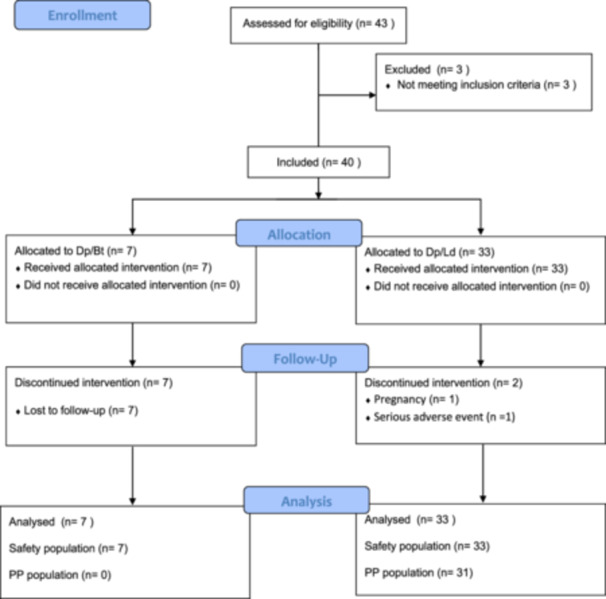

**Abbreviations**
ACQ: asthma control test questionnaire
ADR: adverse drug reaction
AE: adverse event
AIT: allergen immunotherapy
AQLQ: asthma quality of life questionnaire
cSMS: combined symptom and rescue medication score
Dpt/Bt: *Dermatophagoides pteronyssinus* and *Blomia tropicali*
Dpt/Ld: *Dermatophagoides pteronyssinus* and *Lepidoglyphus destructor*
EAACI: European Academy of Allergy and Clinical Immunology
FEV1: forced expiratory volume in the first second
HDM: house dust mite
IMP: investigational medicinal product
PEFR: peak exploratory flow rate
RQLQ: rhinoconjunctivitis quality of life questionnaire
SCIT: subcutaneous allergen immunotherapy
SD: standard deviation
TEAEs: treatment‐emergent adverse events
V(x): visit (x)
VAS: visual analogue scale

## 1 Introduction

House dust mite (HDM) allergy has been estimated to affect 1% to 2% of the world's population, which accounts for 65 to 130 million people [1]. HDM sensitisation plays an important etiological role in patients with allergic respiratory diseases, mainly allergic asthma, allergic rhinitis, and rhinoconjunctivitis [2, 3, 4, 5]. In Spain, the most prevalent HDM causing allergy‐related diseases are *Dermatophagoides pteronyssinus* and *Dermatophagoides farinae* followed by *Lepidoglyphus destructor* and *Blomia tropicalis*, the latter mainly in the Canary Islands [6]. *B. tropicalis* is a clinically relevant species in tropical and subtropical regions and coexists with *D. pteronyssinus*; consequently, the dual sensitisation to both HDMs is common in these regions [7, 8, 9, 10]. *L. destructor* can be found in half of the homes in Northern Spain, where 60% of patients are sensitised to both *L. destructor* and *D. pteronyssinus* [11].

Allergen immunotherapy (AIT) remains the only form of treatment that can modulate the underlying immune mechanisms and induce long‐term allergen tolerance [12, 13]. Clinical trials have demonstrated the efficacy and safety of subcutaneous immunotherapy (SCIT) for allergic asthma and rhinoconjunctivitis [14, 15], which have been confirmed by real‐world studies [16, 17, 18]. Most patients are polysensitized and this has been associated with a higher risk of respiratory allergic diseases and more severe symptoms. In Spain, for instance, data from 2015 showed that around 70% of allergic patients attending allergy clinics were polysensitized [19]. Therefore, the use of allergen mixtures offers practical advantages when treating polyallergic patients.

In this sense, the efficacy of AIT is dose‐dependent, and it is necessary to reach the maintenance dose achieve clinical benefit [20, 21, 22]. High doses of allergen extracts imply a greater risk of adverse reactions [23], but mixtures containing the maximum extract concentrations are needed to meet the therapeutic requirements of polyallergic patients. Therefore, we designed a study to assess the safety and efficacy of two depigmented and polymerized mixtures at maximum concentrations of each mite allergen extract.

Our primary objective was to assess the safety of mixtures of *D. pteronyssinus* and *B. tropicalis* (Dpt/Bt) and of *D. pteronyssinus* and *L. destructor* (Dpt/Ld) allergen extracts in patients with allergic rhinitis or rhinoconjunctivitis with controlled allergic asthma due to sensitisation to these mites, coming from the Canary Islands and Galicia (Northern Spain), respectively.

Secondary objectives included evaluating the efficacy of the intervention in reducing the use of medication, relieving symptoms, and improving quality of life after 2 years of treatment. We also aimed to evaluate the mechanism of action of the two allergen extracts by measuring immunological blood parameters.

## 2 Methods

### 2.1 Study Design and Setting

This was an open‐label, noncontrolled, nonrandomised, phase IIb clinical trial (EudraCT 2014‐000172‐26; 2014) carried out in three hospitals in Spain (University Hospital Nuestra Señora de la Candelaria, Tenerife; University Hospital of Santiago, Santiago de Compostela; and Hospital Lucus‐Augusti, Lugo) between September 2014 and May 2018. The study was approved by the Independent Ethics Committee of University Hospital Nuestra Señora de la Candelaria and by the Ethical Committee of Research of Galicia. The study was undertaken in accordance with the Declaration of Helsinki [24]. Patient data confidentiality was preserved, and their dissociation was ensured, duly informing participating subjects as provided in Spanish Organic Law 15/1999, of 13 December, and in Royal Decree 1720/2007 [25], in accordance with the Directive 95/46/EC of the European Parliament and of the Council of 24 October 1995 [26]. Candidates provided their written informed consent before being enrolled in the study.

### 2.2 Participants

We included patients between 18 and 70 years old, with perennial, moderate‐to‐severe allergic rhinitis or rhinoconjunctivitis for at least 1 year, with controlled asthma (according to the 2014 GINA guidelines) [27], and positive IgE‐mediated sensitisation against either *D. pteronyssinus* and *B. tropicalis* or *D. pteronyssinus* and *L. destructor* (positive skin prick test ≥ 3 mm and specific IgE ≥ 0.7 KU/l). Additionally, patients were required to have a forced expiratory volume in the first second ≥ 80%.

The main exclusion criteria included any contraindications for AIT, previous history of anaphylaxis, acute or chronic infectious conjunctivitis, chronic inflammatory or infectious airway diseases, diseases of the immune system, chronic urticaria, moderate‐to‐severe atopic dermatitis (SCORAD value > 30) [28], malignant disease within 5 years before the study start, and hospital admission due to asthma exacerbations within 1 year before the study start.

### 2.3 Intervention

Patients received specific SCIT with mixtures of either investigation medicinal product (IMP), according to their sensitisation. Both IMPs were mixtures of depigmented allergen extracts at maximum concentrations, polymerised with glutaraldehyde, of either *D. pteronyssinus* and *B. tropicalis* (concentration: 100/1000 DPP/mL) or *D. pteronyssinus* and *L. destructor* (concentration: 100/100 DPP/mL) adsorbed in aluminum hydroxide (commercialized as Depigoid DUO).

The SCIT was administered in two phases: first, a rush build‐up phase on the first day of administration as two subcutaneous injections of 0.2 mL and 0.3 mL with a 30‐min interval between them, in alternate arms. Second, a maintenance phase from the second day of administration onwards (4 weeks after the initial rush build‐up phase), in which the patient received a monthly 0.5 mL subcutaneous injection, as recommended by the manufacturer (LETI Pharma S.L.U., Madrid, Spain), for a total of 48 months.

### 2.4 Variables and Assessments

**Figure 1:**
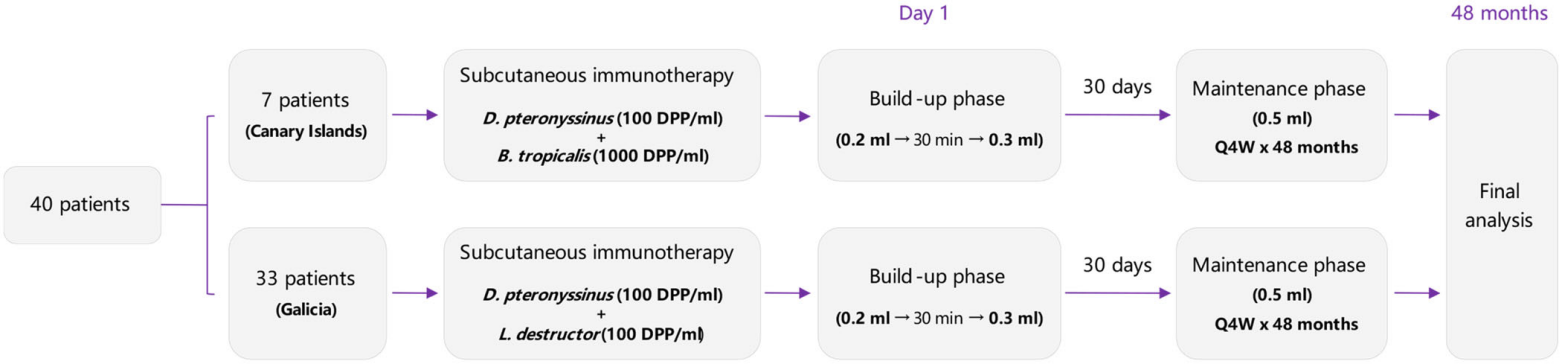
Study design. Q4W: once every 4 weeks.

Eight visits were scheduled, divided into the screening visit, the safety period, and the efficacy follow‐up period. A diagram of the study design and visits is shown in Figure 1.

At V1, the patients' medical history was recorded, a skin prick test and a specific IgE test to determine their IgE‐mediated sensitisation were performed, and their blood samples were analysed. At all visits, patients underwent a physical examination, and their vital signs were monitored before IMP administration. Patients underwent lung function tests (a peak exploratory flow rate [PEFR]) at all visits except at V4.

During the safety period, we registered adverse events (AEs), including systemic and local adverse reactions (ARs), and patients' blood samples were collected and analysed (blood counts and biochemistry) at V4. Immediate ARs were those occurring within the first 30 min after IMP administration; otherwise, they were considered delayed ARs. We classified ARs according to the 2006 European Academy of Allergy and Clinical Immunology criteria (EAACI) [29].

During the efficacy follow‐up period, all AEs were recorded. Additionally, the combined symptom and rescue medication score (cSMS, Supporting Information S1: Table S1) [30] was collected in a diary given to patients at V1, V5, V6, and V7 and filled in the subsequent 15 days. For V8, the diary was given to the patient 4 weeks earlier, at the last administration visit. At V6 and V8 blood samples were collected and analysed. The asthma control test questionnaire (ACQ, validated in Spanish [31], score: from 0 [well‐controlled] to 6 [extremely poor‐controlled]), the asthma quality of life questionnaire (AQLQ, validated in Spanish [32], score: from 1 [severely impaired] to 7 [not impaired at all]), and the rhinoconjunctivitis quality of life questionnaire (RQLQ, validated in Spanish [33], score: from 0 [not impaired at all] to 6 [severely impaired]), were completed at V1, V5, V6, V7, and V8. At these same visits, the patients indicated their perception of disease severity on a 100‐mm horizontal visual analogue scale (VAS), ranging from “absence of symptoms” (0) to “very severe symptoms” (100) [34].

For the safety analysis, we used the safety population, defined as all patients who received at least one dose of either IMP. For the efficacy analysis, we used the per‐protocol population, which encompassed all patients included in the study and without major protocol deviations who received all doses of the IMP and presented at least one valid evaluation for the cSMS.

### 2.5 Statistical Analysis and Sample Size

Continuous variables were presented with the number of observations, mean, and standard deviation (SD), or median and interquartile range (IQR) (Q1, Q3) or range (min, max), when normality could not be assumed. Categorical variables were presented with frequencies and percentages. For the primary safety endpoint, we used the binomial exact test for the interference analysis to compare proportions. The paired *t*‐test with a bilateral significance of 5% was used for normally distributed data, while the non‐parametric Wilcoxon test was used when normality could not be assumed.

To determine the sample size, we used Simon's optimal two‐stage design methodology (with a type I error of 0.05 and a statistical power of 90%). In both IMP groups, if the number of patients with grade ≥ 2 systemic reactions (according to EAACI classification) did not exceed a predefined limit ( ≥ 4) in the initial 18 subjects, the study was expected to continue with the inclusion of 16 additional patients up to a total of 34 subjects. Conversely, if among these 18 initial subjects, the number of subjects with systemic reactions was above the predefined limit the study would end prematurely. This sample size was equivalent to a statistical power of 85% to detect a 2‐point drop in the cSMS, assuming an SD of 5, and using a paired *t*‐test with a bilateral significance of 5%.

## 3 Results

### 3.1 Demographic and Clinical Characteristics of Patients

**Figure 2:**
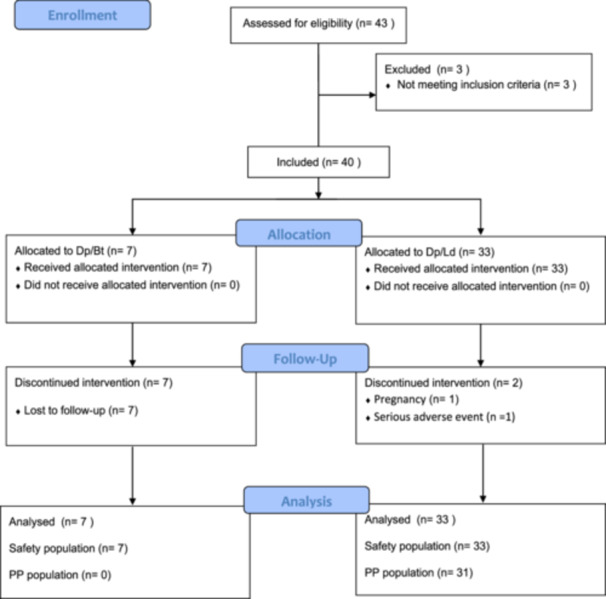
Patient enrolment flow chart. Dpt/Bt: Allergen extract mixture of *Dermatophagoides pteronyssinus* and *Blomia tropicalis* 100/1000 DPP/mL; Dpt/Ld: Allergen extract mixture of *Dermatophagoides pteronyssinus* and *Lepidoglyphus destructor* 100/100 DPP/mL.

Of the 40 patients included in the study, seven were allocated to the Dpt/Bt arm and 33 to the Dpt/Ld arm, based on their sensitisation profile (Figure 2). The baseline characteristics of our cohort are summarised in Table 1. In both groups, most patients were female (*n* = 6; [85.7%] in Dpt/Bt and *n* = 20; [60.6%] in Dpt/Ld) and had never smoked (*n* = 4; [57.1%] in Dpt/Bt and *n* = 21; [63.6%] in Dpt/Ld). The median age was 29.0 years old [range: 18, 55] in the Dpt/Bt group and 26.4 years old [range: 18, 62] in the Dpt/Ld group.

**Table 1:** Demographic and clinical characteristics of study participants.

	Overall	*D. pteronyssinus* + *B. tropicalis* a	*D. pteronyssinus* + *L. destructor* b
	(*N* = 40)	(*N* = 7)	(*N* = 33)
**Sex, *n* (%)**			
Female	26 (65.0)	6 (85.7)	20 (60.6)
Male	14 (35.0)	1 (14.3)	13 (39.4)
**Age, median (range)–years**	26.5 (18; 62)	29.0 (18; 55)	26.4 (18; 62)
**BMI, mean (SD)–kg/m** ^**2**^	25.2 (4.9)	24.3 (4.5)	25.3 (5.0)
**Age of allergy onset, median (range) – years**			
Asthma^c^	10.0 (1; 51)	10.0 (3; 51)	10.0 (1; 46)
Rhinitis/rhinoconjunctivitis^c^	13.0 (1; 46)	5.0 (3; 40)	13.0 (1; 46)
**Mite‐specific IgEs, median (Q1; Q3) ‐ kU/L**			
*D. pteronyssinus*	47.3 (15.2; 78.9)	25.4 (13.4; 100)	48.6 (19.8; 77.3)
*L. destructor*	17.6 (8.9; 30.8)	N/A	17.6 (8.9; 30.8)
*B. tropicalis*	6.6 (0.9; 28.0)	6.6 (0.9; 28.0)	N/A
**Skin prick test, median wheal diameter (Q1; Q3) – mm** d			
*D. pteronyssinus*	11.5 (8.5; 13.0)	11.5 (7.0; 13.0)	11.5 (9.0; 13.0)
*L. destructor*	12.0 (8.5; 15.0)	5.5 (3.0; 8.5)	12.5 (11.0; 15.5)
*B. tropicalis*	5.5 (1.5; 8.5)	7.5 (5.0; 8.0)	5.0 (0.0; 8.5)
**Atopic dermatitis, *n* (%)** e	7 (17.5)	2 (28.6)	5 (15.2)
**Food allergy, *n* (%)**	5 (12.5)	2 (28.6)	3 (9.1)
**Family history of atopy, *n* (%)**			
Mother	4 (10.0)	2 (28.6)	2 (6.1)
Father^f^	8 (20.5)	2 (33.3)	6 (18.2)
Siblings^f^	13 (33.3)	1 (16.7)	12 (36.4)

Abbreviations: BMI, body mass index; d, wheal shorter diameter; D, wheal longest diameter; N/A, Not applicable; Q1, first quartile; Q3, third quartile; SD, standard deviation.

^a^ Allergen extract mixture of *Dermatophagoides pteronyssinus* (100 DPP/mL) and *Blomia tropicalis* (1000 DPP/mL).

^b^ Allergen extract mixture of *Dermatophagoides pteronyssinus* (100 DPP/mL) and *Lepidoglyphus destructor* (100 DPP/mL).

^c^ All patients had asthma and rhinitis or rhinoconjunctivitis.

^d^ Mean wheal diameter was calculated as D + d/2, with “D” indicating the largest diameter of the wheal and “d” indicating the largest diameter orthogonal to D.

^e^ Mean (SD) SCORAD 3.24 (6.8).

^f^ Overall (*N* = 39) and *D. pteronyssinus* + *B. tropicalis* (*N* = 6).

### 3.2 Safety of Subcutaneous Immunotherapy

The extent of exposure in the safety period was as follows: in the Dpt/Bt group, three patients (43%) received five administrations; three (43%), four administrations; and one (14%), three administrations; in the Dpt/Ld group, 31 patients (94%) received six administrations; one (3%), four administrations; and one (3%), three administrations. A total of 40 patients from both arms (7 in the Dpt/Bt group and 33 in the Dpt/Ld group) were considered the safety population. None of the 40 subjects experienced immediate or delayed systemic grade ≥ 2 reactions (EAACI classification) regardless of the IMP received. No subjects died during the study period.

#### 3.2.1 Local and Systemic Reactions

In the Dpt/Bt group, one patient (14%) experienced one grade 0 systemic reaction, and in the Dpt/Ld group, five patients (15%) experienced six grade I (mild) and one grade 0 systemic reactions (Table 2). Regarding local reactions, in the Dpt/Bt group, three patients (43%) experienced 3 mild immediate reactions and 2 mild delayed reactions. In the Dpt/Ld group, seven patients (21%) experienced 1 mild immediate reaction and 7 delayed reactions; 6 of which were mild and 1 moderate. No patients were withdrawn from the study because of systemic or local reactions, including those experienced during the rush build‐up phase. Two patients (5%) discontinued AIT permanently because of thrombophlebitis (*n* = 1) and asthma‐worsening respiratory infection (*n* = 1), unrelated to IMP administration.

**Table 2:** Patients in the safety population with immediate or delayed systemic or local reactions by treatment.

	Overall	*D. pteronyssinus* + *B. tropicalis* a, b	*D. pteronyssinus* + *L. destructor* a, c
	(*N* = 40)	(*N* = 7)	(*N* = 33)
**Systemic reactions**			
Number of patients, *n* (%)	6 (15.0)	1 (14.0)	5 (15.0)
Number of reactions, *n* d	8	1	7
Grade 0, *n* (%)	2 (25.0)	1 (100.0)	1 (14.0)
Grade I (mild), *n* (%)	6 (75.0)	0 (0.0)	6 (86.0)
**Local reactions**			
Number of patients, *n* (%)	10 (25.0)	3 (43.0)	7 (21.0)
Number of reactions, *n*	14	5	9
Immediate, *n*	4	3	1
Delayed, *n*	10	2	8
Wheal size immediate reaction, *n* (%)			
Mild (0–5 cm)	4 (100.0)	3 (100.0)	1 (100.0)
Wheal size delayed reaction, *n* (%)			
Mild (0–10 cm)	8 (80.0)	2 (100.0)	6 (75.0)
Moderate (10.1–15 cm)	2 (20.0)	0 (0.0)	2 (25.0)

^a^ According to the European Academy of Allergy and Clinical Immunology (EAACI) classification.

^b^ Allergen extract mixture of *Dermatophagoides pteronyssinus* (100 DPP/mL) and *Blomia tropicalis* (1000 DPP/mL).

^c^ Allergen extract mixture of *Dermatophagoides pteronyssinus* (100 DPP/mL) and *Lepidoglyphus destructor* (100 DPP/mL).

^d^ Data for one systemic reaction was missing.

#### 3.2.2 Adverse Events

In the safety population, 39 patients (97.5%) experienced at least one AE; 7 (100%) of them in the Dpt/Bt group and 32 (97.0%) in the Dpt/Ld group. Of the 287 reported AEs, 279 (97.2%) were treatment‐emergent adverse events (TEAEs) and were mostly mild (*n* = 256, 89.2%) and moderate (*n* = 30, 10.5%), although 1 (0.3%) was severe. Eight (2.9%) serious AEs were reported in four subjects, including ligament sprain, meniscus injury, thrombophlebitis, asthma, bronchospasm, and asthma crisis. All serious AEs were TEAEs. Of the 279 TEAEs, 22 (7.8%) were adverse drug reactions (ADRs) (i.e., TEAE related to the IMP) experienced by 13 (32.5%) subjects and were mostly mild (*n* = 20, 90.9%). The most frequent ADRs were pruritus (*n* = 4, 18.2%) and skin reaction (*n* = 3, 13.6%) (Table 3). The most frequent TEAEs are shown in Supporting Information S1: Table S2.

**Table 3:** Summary of ADRs by system organ class, preferred term and intensity.

	Overall^a^	Mild ADR	Moderate ADR
	(*N* = 22)	(*N* = 20)	(*N* = 2)
**Skin and subcutaneous tissue disorders, *n* (%)**	11 (50.0)	9 (45.0)	2 (100.0)
Pruritus	4 (18.2)	4 (20.0)	0 (0.0)
Skin reaction	3 (13.6)	1 (5.0)	2 (100.0)
Erythema	2 (9.1)	2 (10.0)	0 (0.0)
Skin induration	1 (4.5)	1 (5.0)	0 (0.0)
**General disorders and administration site conditions, *n* (%)**	6 (27.3)	6 (30.0)	0 (0.0)
Local reaction	3 (13.6)	3 (15.0)	0 (0.0)
Injection site pruritus	2 (9.1)	2 (10.0)	0 (0.0)
Fatigue	1 (4.5)	1 (5.0)	0 (0.0)
**Respiratory, thoracic and mediastinal disorders, *n* (%)**	3 (13.6)	3 (15.0)	0 (0.0)
Asthma	2 (9.1)	2 (10.0)	0 (0.0)
Dyspnoea	1 (4.5)	1 (5.0)	0 (0.0)
**Infections and infestations, *n* (%)**	1 (4.5)	1 (5.0)	0 (0.0)
Rhinitis	1 (4.5)	1 (5.0)	0 (0.0)
**Investigations, *n* (%)**	1 (4.5)	1 (5.0)	0 (0.0)
Pulmonary function test decreased	1 (4.5)	1 (5.0)	0 (0.0)

Abbreviation: ADR, adverse drug event.

^a^ No severe ADRs were reported.

### 3.3 Efficacy Analysis

Since all patients in the Dpt/Bt group were lost to follow‐up, did not attend appointments, and could not be reached by phone or letter (Figure 2), efficacy results were only analysed in the per‐protocol population of the Dpt/Ld group (*n* = 31). At V8 (24 months), 29 patients (93.5%) were still on treatment.

#### 3.3.1 Combined Symptom and Rescue Medication Score

**Figure 3:**
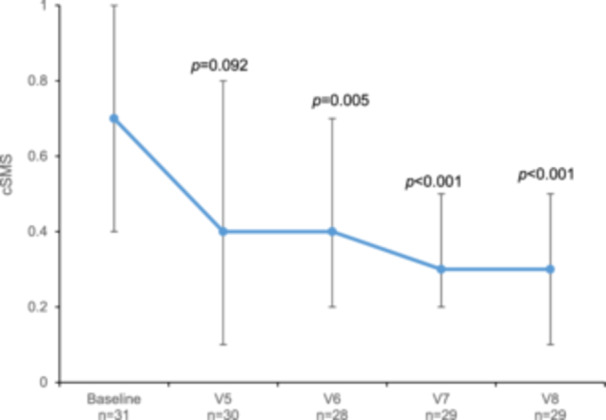
Median combined symptom and rescue medication scores (cSMS) from baseline to the last visit in the Dpt/Ld group (per protocol population). Vertical lines represent the interquartile range (Q1, Q3). *p* values correspond to differences between baseline and each visit and were calculated with paired t‐test.

The median cSMS gradually decreased throughout the study, with significant differences at V6 (*p* = 0.005), V7 (*p* < 0.001), and V8 (*p* < 0.001) compared to baseline (Figure 3). The number of patients with a decreased cSMS with respect to baseline gradually increased from V5 (*n* = 20, 63%) to V8 (*n* = 26, 84%).

#### 3.3.2 Nasal, Ocular, and Bronchial Symptom Scores

**Figure 4:**
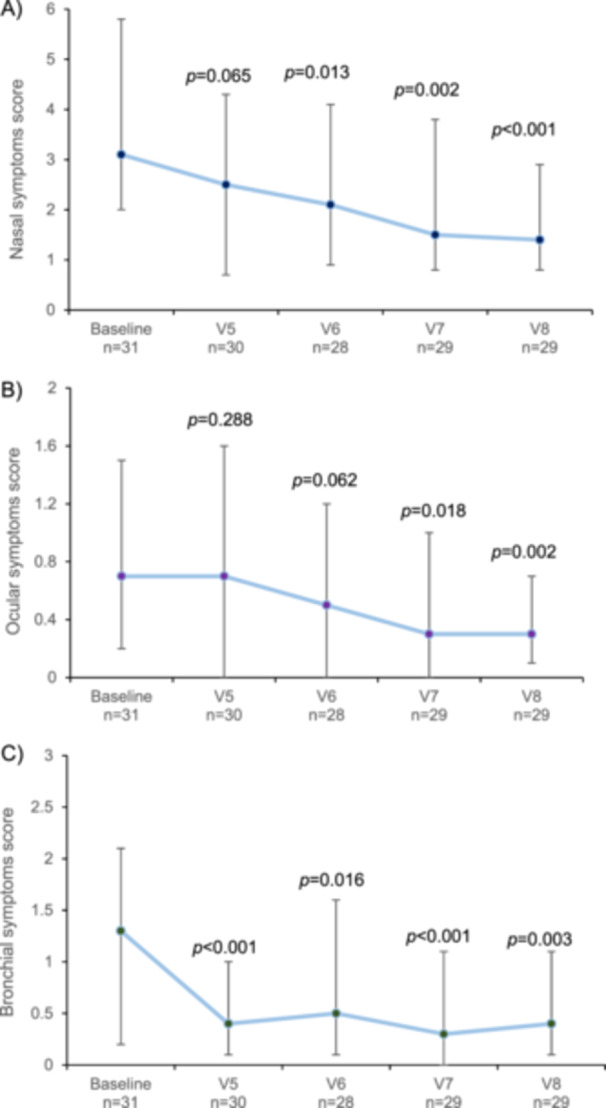
Median symptom scores from baseline to the last visit in the Dpt/Ld group (per protocol population): (A) nasal, (B) ocular, and (C) bronchial symptoms. Vertical lines represent the interquartile range (Q1, Q3). *P*‐values correspond to differences between baseline and each visit, and were calculated with paired *t*‐test.

All symptom scores gradually decreased between baseline and the follow‐up period (from V5 to V8) (Figure 4). Changes were statistically significant for nasal symptom scores between baseline and V6 (*p* = 0.013), V7 (*p* = 0.002), and V8 (*p* < 0.001). Changes were statistically significant for ocular symptom scores between baseline and V7 (*p* = 0.018) and V8 (*p* = 0.002). Changes were statistically significant for bronchial symptom scores between baseline and V5 (*p* < 0.001), V6 (*p* = 0.016), V7 (*p* < 0.001), and V8 (*p* = 0.003). At the end of the follow‐up period (i.e., V8 at 24 months), 22 patients (76%) showed a decrease in nasal symptoms, 21 (72%) in ocular symptoms, and 20 (69%) in bronchial symptoms with respect to baseline. Similar results were obtained for the rescue medication with significant reductions between baseline and V6 (*p* = 0.020), V7 (*p* = 0.009), and V8 (*p* < 0.001).

#### 3.3.3 Patient‐Reported Outcomes

**Figure 5:**
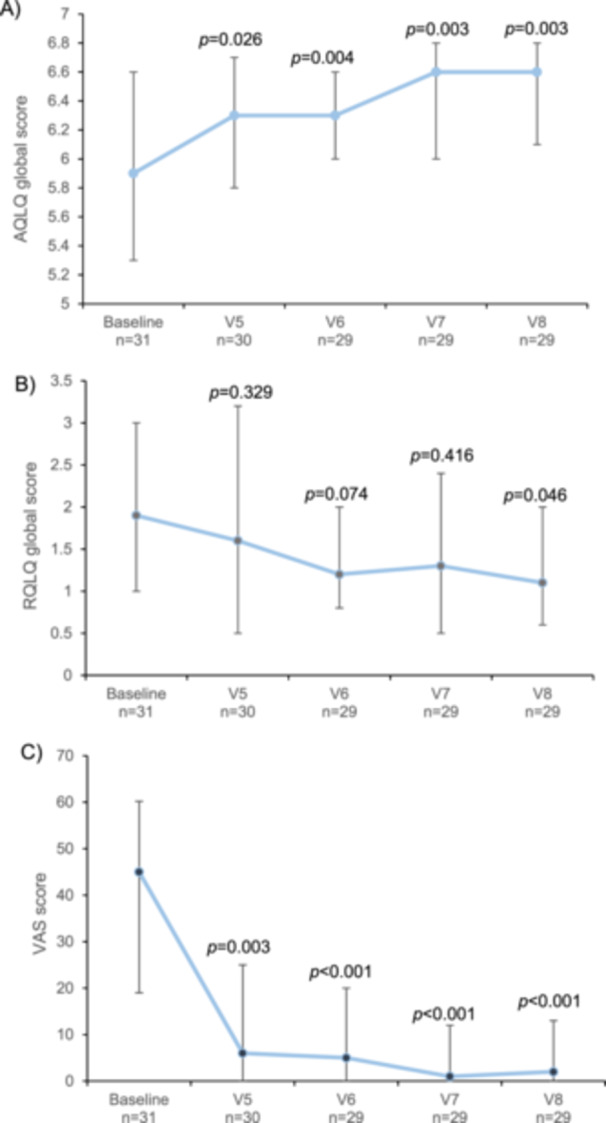
Median patient‐reported outcomes: (A) Asthma quality of life questionnaire (AQLQ) global score, (B) Rhinoconjunctivitis quality of life questionnaire (RQLQ) global score, and (C) Visual analogue scale (VAS) scores from baseline to the last visit in the Dpt/Ld group (per protocol population). Vertical lines represent the interquartile range (Q1, Q3). Scale is 5 to 7 on graph A. *p* values correspond to differences between baseline and each visit and were calculated with paired *t*‐test.

The median (IQR) ACQ scores were 0.571 (0.285, 0.858) at baseline, and significantly decreased to 0.286 (0.143, 0.285) at V8 (*p* < 0.001). The global score of the AQLQ increased significantly at V5 (*p* = 0.026), V6 (*p* = 0.004), V7 (*p* = 0.003), and V8 (*p* = 0.003) with respect to baseline. In contrast, the global score of the RQLQ remained stable throughout the follow‐up period, but a significant decrease was finally reached at V8 (*p* = 0.046) compared to baseline (Figure 5). Scores of the VAS significantly decreased at V5 (*p* = 0.003), V6 (*p* < 0.001), V7 (*p* < 0.001), and V8 (*p* < 0.001) compared to baseline (Figure 5).

#### 3.3.4 Immunological Parameters

**Figure 6:**
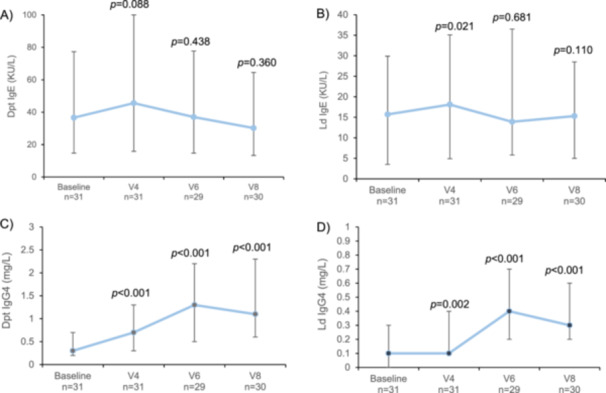
Median levels of (A) Dpt IgE (KU/L), (B) Ld IgE (KU/L), (C) Dpt IgG4 (mg/L), and (D) Ld IgG4 (mg/L) from baseline to the last visit in the Dpt/Ld group (per protocol population). Vertical lines represent the interquartile range (Q1, Q3). *p* values correspond to differences between baseline and each visit, and were calculated with paired *t*‐test.

Specific IgE (sIgE) levels of *D. pteronyssinus* remained unchanged throughout the study, whereas those of *L. destructor* experienced a transient, significant increase at V4 (*p* = 0.021). IgG4 levels of *D. pteronyssinus* and *L. destructor* significantly increased from baseline throughout V4, V6, and V8 (Figure 6).

#### 3.3.5 Lung Function

In the Dpt/Ld group, the mean PEFR (SD), measured before and 30 min after each AIT administration, significantly decreased after the first dose at V2 from 455.3 L/min (98.1%) to 446.0 L/min (94.2%; *p* = 0.018), and at V3 from 475.5 L/min (108.7%) to 459.8 L/min (105.0%; *p* = 0.012). However, PEFR levels always remained within the normal range.

## 4 Discussion

SCIT treatment with mixtures of Dpt/Bt and Dpt/Ld at maximum concentrations showed a favourable safety profile, with no immediate or delayed systemic reactions of grade ≥ 2. Only a few patients showed mild systemic reactions and mild‐to‐moderate local reactions. Most AEs were mild and moderate TEAEs, of which only a few were ADRs. SCIT treatment with a mixture of Dpt/Ld reduced cSMS from V6 onwards and gradually decreased all symptom and medication scores. After 2 years of treatment, patients improved their control and perception of asthma and rhinoconjunctivitis, as well as their quality of life.

Regarding safety, the two mixtures of Dpt/Bt and Dpt/Ld extracts at maximum concentrations showed a favourable profile. Severe systemic reactions in patients with asthma, especially noncontrolled asthma, treated with subcutaneous AIT are a matter of concern among specialists [35, 36]. Notably, all patients included in our study had asthma, but no severe adverse reactions were detected and their PEFR was always within the normal range. Interestingly, current asthma and allergic rhinitis management guidelines have included AIT as an effective treatment for allergic diseases caused by inhaled allergens in eligible patients [37, 38]. In addition, recent guidelines on allergic rhinoconjunctivitis have stressed the pivotal role of AIT as the only treatment with a disease‐modifying effect [39].

In the group receiving Dpt/Ld, the cSMS significantly improved from 12 months after starting treatment and continued improving at the end of the study, 2 years after initiating AIT. Of note, the cSMS is recommended by the EAACI as the primary efficacy outcome in AIT trials [30], and a 15% to 20% reduction in the cSMS is generally accepted as clinically relevant [40, 41, 42]. In our study, the decrease compared to the baseline in the cSMS was greater than that threshold already 6 months after treatment initiation and, even more important, the cSMS continued to decrease until the end of the study, which supports the clear benefit of this therapy. Although we did not conduct a cost‐effectiveness analysis, the significant decrease in the use of costly rescue medication could also be considered among the positive effects of this therapeutical approach.

Similarly, patients' symptoms also improved gradually, with significant differences from baseline after 12–18 months of treatment for nasal and ocular symptoms. Notably, bronchial symptoms showed an improvement as early as 6 months after receiving AIT injections, with most patients experiencing a decrease in all symptoms after 2 years. Interestingly, treatment adherence was very high 2 years after AIT initiation, as nearly all patients remained on treatment at the end of the study period, further supporting the positive safety and efficacy profile of Dpt/Ld mixture.

Regarding patient‐reported outcomes, asthma questionnaire results showed an improvement in disease control and patient quality of life after 2 years. Our results were in line with some previous studies on SCIT [43, 44, 45], highlighting its benefits for the patient. Similarly, the quality of life of patients with rhinoconjunctivitis improved after 2 years of treatment, which was also consistent with previous reports [15]. Importantly, patients' perception of their disease improved from 6 months after AIT initiation. Of note, patients started the study with a severely impaired QoL affecting their work and social life, and the improvement achieved with AIT treatment could also have had a positive impact on adherence, as shown elsewhere [46].

The mechanism of action of AIT involves a transient increase in IgE levels after treatment initiation and a decrease to pretreatment levels during the maintenance phase. At the same time, in effective AIT treatments, antibodies of the IgG4 subclass increase, as they have the potential to influence clinical response to the allergen by modulating immune response since they act as blocking antibodies [47, 48]. These results show that Dpt/Ld mixture has an immunological response given that IgG4 levels of *D. pteronyssinus* and *L. destructor* increased from 5 weeks after the rush build‐up phase, with those of IgE remaining mostly stable.

Mixing different allergens for AIT is controversial, with some authors claiming its clinical relevance [49], while others being wary of the dilution effect and potential allergen degradation [37]. In fact, the European Medicines Agency (EMA) has recommended only mixing allergens, represented by their sources, from homologous groups [50, 51]. In this sense, our IMPs complied with this regulation. However, the EMA also specifies that, when mixing allergens from heterologous groups, a justification is required. This is the reality of allergic patients, especially in Europe, where polysensitization reaches 70% in most Mediterranean countries and over 50% in Northern Europe [19, 52, 53]. Therefore, treatment with mixtures at maximum concentrations would be, in some cases, preferable over two parallel single AITs since, by reducing the number of injections, it simplifies the regimen, cutting down the cost and improving adherence [54]. Additionally, studies showing non‐degradation and potency have been previously published to justify these mixtures [1].

Currently, most available subcutaneous and sublingual AITs combine the most common HDMs, i.e., *D. pteronyssinus* and *D. farinae* [14, 15]. In contrast, the IMPs studied here combined a common HDM (*D. pteronyssinus*) with another important, but less common, mite (*B. tropicalis* or *L. destructor*), and could thus be considered profile‐targeted treatments addressing specific patient needs. Furthermore, although *B. tropicalis* and *L. destructor* are less prevalent mites, their presence is not only restricted to Spain, since reports of cities in Poland [55], Portugal [56], Scandinavia [57], Chile [58], Colombia [59], Mexico [60], and Venezuela [61] have also raised the issue on allergic problems related to these mites.

Altogether, SCIT Dpt/Ld proved to be safe, considering it carried both allergens at their maximum concentrations, and was clearly effective in treating patients with a clinically relevant sensitization to either *D. pteronyssinus* or *L. destructor*, an outcome supported by previous studies on mixed allergen extracts [62, 63].

### 4.1 Strengths and Limitations

This study had a 2‐year follow‐up period, which contrasts with most similar studies designed for up to 1 year [14]. This longer follow‐up allowed us to reach significant results in some parameters that could have otherwise been overlooked and is closer to the 3 years of AIT treatment [39]. In addition, although this was a clinical trial, we included real‐world patients, which could favour the introduction of Dpt/Bt and Dpt/Ld SCIT into the routine clinical practice.

To the best of our knowledge, there are no previous clinical data directly supporting the safety and efficacy of immunotherapy with *L. destructor* allergen extracts. However, our results should be interpreted in light of their limitations, mainly the lack of a control group, which makes it difficult to draw direct conclusions about the efficacy of SCIT Dpt/Ld compared to standard care or placebo. Nevertheless, we understand that single‐group studies can provide information about causal treatment effects by extrapolating expected outcomes in the missing untreated arm. As others have suggested, single‐group studies rely on implicit or historical comparisons as a surrogate for an ideal comparison group. In this regard, the efficacy and safety of *D. pteronyssinus* depigmented allergen extract in patients with allergic asthma was demonstrated in a randomized, double‐blind, placebo‐controlled trial [64]. In this study, the active group showed a significant increase in PD_20_FEV_1_ compared to placebo (*p* = 0.0029). Nineteen patients in the active group versus 10 in the placebo group required more than twice the initial amount of allergen extract to achieve a positive bronchial provocation test (*p* = 0.0293), whereas seven patients in the placebo group versus one in the active group required less than half the amount (*p* = 0.0137). The active group also had a median reduction in symptom scores of 91.5% compared to an 86% increase in the placebo group. Medication scores decreased very differently in the active and control groups, by 56% and 11.4%, respectively. In addition, significant differences in quality of life were observed between the two groups at the end of the study (*p* = 0.0234). Although our results are preliminary, further research is warranted to provide more robust data that may ultimately provide a clearer understanding of the drug's therapeutic potential.

In allergen immunotherapy research, practical challenges frequently emerge due to small patient populations for rare allergens, making it difficult to enroll enough participants for both control and treatment groups. Regulatory guidelines acknowledge these difficulties and may allow single‐arm trials when the therapy meets significant unmet medical needs or addresses rare conditions [65]. Nevertheless, the improvement observed in clinical objective variables such as lung function and immunological parameters, is related to a genuine effect of immunotherapy rather than a placebo effect.

Other limitations in our study were the loss of the Dpt/Bt arm for the efficacy analysis and the overall small study population. In this sense, although the statistical power was sufficient to detect changes in efficacy variables between visits, some infrequent ADRs could have gone undetected.

## 5 Conclusions

The treatment of patients with allergic rhinitis or rhinoconjunctivitis and controlled asthma with mixtures of Dpt/Bt and Dpt/Ld at maximum concentrations showed a favourable safety profile. Treatment with Dpt/Ld effectively reduced the cSMS and improved nasal, ocular, and bronchial symptoms 1 year after SCIT initiation. Importantly, the control, quality of life, and perception of asthma and rhinoconjunctivitis improved during the study and remained stable after 2 years. Our results warrant further studies on mixed mites AIT with prevalent and non‐prevalent allergens in larger population settings and with a focus on cost‐effectiveness.

## Author Contributions

All authors included patients and contributed to the writting and review of this manuscript.

## Conflicts of Interest

Eva Nieto and Monica Ruiz‐Garcia work for LETI Pharma. All other authors declare no competing interests.

## Supporting information

Supporting information.

## Data Availability

The authors have nothing to report.
