# Supplementary material for: Clinical Trial With a Depigmented, Polymerized Mite Mixture Extract at Maximum Concentrations
Source: Immun Inflamm Dis. 2024 Dec 19;12(12):e70090. doi: 10.1002/iid3.70090 (PMC11656404; doi:10.1002/iid3.70090)
Supplement: Supplementary file 1 — Supporting information. [file IID3-12-e70090-s001.docx]

| **Supplementary Table S1. Scoring of the combined symptom and rescue medication score** | | | | |
| --- | --- | --- | --- | --- |
|  | 0 | 1 | 2 | 3 |
| Severity of the symptom^a^ | No symptom | Mild | Moderate | Severe |
| Rescue medication score^b^ | No medication | Non-sedating oral or topical H1A for eyes or nose | INC with or without H1A | Oral corticosteroids with or without INC and with or without H1A |
| ^a^A score was assigned to the severity of nasal (sneezing, hydrorrhea, nasal obstruction), ocular (ocular pruritus and lacrimation), and bronchial (cough, wheezing, and dyspnoea) symptoms. Total score: from 0 to 9.  ^b^Total score: from 0 to 3.  H1A: H1 antihistamines; INC: Intranasal corticosteroids. | | | | |

**Supplementary Figures**


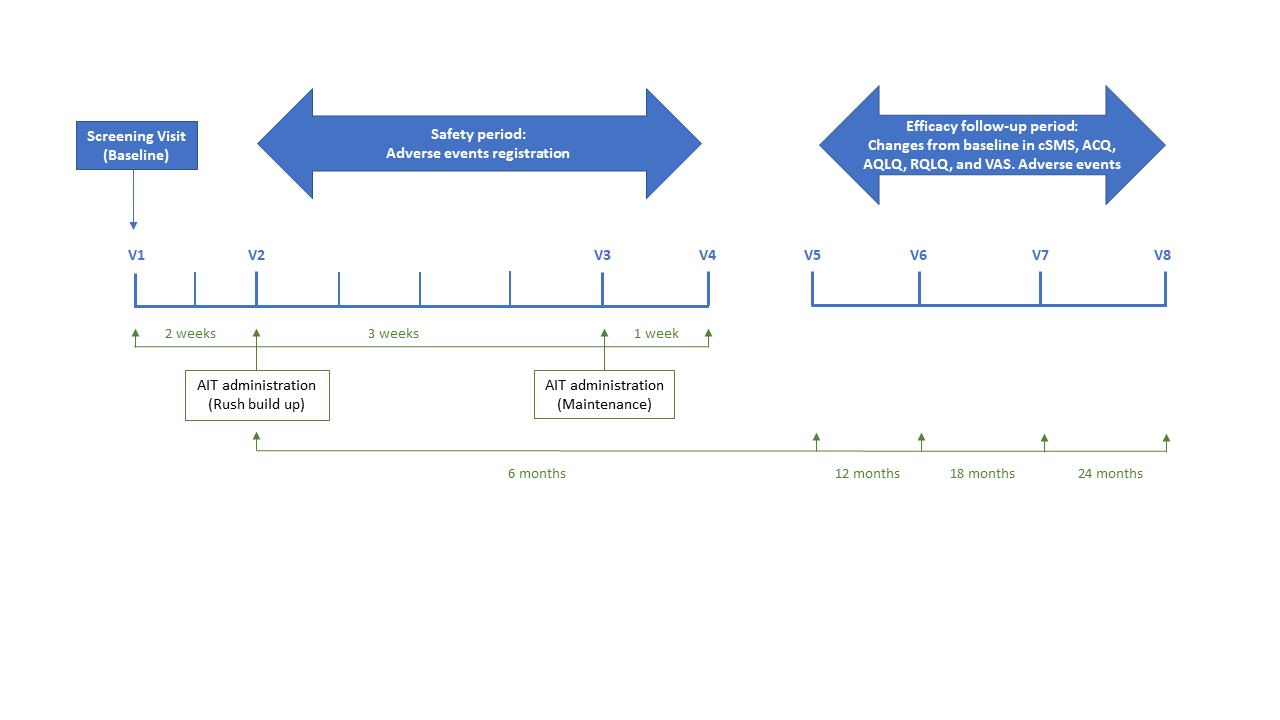


**Figure S1.** Diagram of the study design and visits.

ACQ, asthma control test questionnaire; AQLQ, asthma quality of life questionnaire; cSMS, combined symptoms and medication score; RQLQ, rhinoconjunctivitis quality of life questionnaire; VAS, visual analogue scale.

A)
